# The pararectus approach for internal fixation of acetabular fractures involving the anterior column: evaluating the functional outcome

**DOI:** 10.1007/s00264-018-4148-8

**Published:** 2018-09-14

**Authors:** Christian von Rüden, Lisa Wenzel, Johannes Becker, Andreas Thannheimer, Peter Augat, Alexander Woltmann, Volker Bühren, Mario Perl

**Affiliations:** 1 Department of Trauma Surgery, BG Trauma Centre Murnau, Professor Küntscher Str. 8, 82418 Murnau, Germany; 2 Institute of Biomechanics, BG Trauma Centre Murnau, Murnau, Germany; 3 Institute of Biomechanics, Paracelsus Medical University, Salzburg, Austria

**Keywords:** Acetabular fracture, Quadrilateral plate, Pararectus approach, Stoppa approach, Ilioinguinal approach, Merle d’Aubigné, Lower extremity functional scale, WOMAC, SF-36, Outcome

## Abstract

**Introduction:**

Aim of this retrospective analysis of prospectively collected data was to evaluate the functional mid-term outcome two years after open reduction and internal fixation of acetabular fractures involving the anterior column with affection of the quadrilateral plate using the pararectus approach on a large cohort.

**Method:**

Fifty-two patients (12 female, 40 male) with a median age of 55 (range 18–90) years and displaced acetabular fractures involving the anterior column were surgically treated in a single level I trauma centre between July 2012 and February 2016 using the pararectus approach. Thirty-four patients (8 female and 26 male) with a median age of 58 (range 20–85) years were available for complete clinical follow-up at regular intervals, finally 24 months post-operatively. Functional outcome was evaluated according to modified Merle d’Aubigné score, Lower Extremity Functional Scale, WOMAC, and SF-36.

**Results:**

Range of time between trauma and surgical treatment was three (range 0–19) days. Operation time was 140 (range 60–240) minutes, and duration of hospital treatment was 19 (range 7–38) days. Functional results in 34 patients available for final follow-up demonstrated 68 points (median; range 39–80) according to the Lower Extremity Functional Scale, 6% according to the WOMAC (mean; SD ± 14.5%), and 69% (mean; SD ± 20.1%) according to the SF-36. The modified Merle d’Aubigné score was excellent in 22 patients, good in eight patients, and fair in four patients.

**Discussion/conclusion:**

Based on the good to excellent functional mid-term follow-up results of this study, the pararectus approach can be recommended as sufficient alternative single access to address displaced acetabular fractures involving the anterior column, independent of patients’ age.

## Introduction

The gold standard approach for open reduction and internal fixation of acetabular fractures involving the anterior column was the ilioinguinal approach introduced by Letournel [1]. In the literature, 40–80% of anatomically reduced results using post-operative conventional X-rays were described [2, 3]. Within the last 30 years incidence of acetabular fractures even in the elder population has been almost doubled, and the complexity of these fractures has increased [4]. In the same period of time, significantly more fractures involving the anterior column, dislocations of the quadrilateral plate or impaction of the acetabular dome occurred [4]. In a large retrospective analysis, age over 65 years, initial fracture displacement of more than 2 mm, and fracture pattern involving the anterior column were defined to be important predictive factors for fixation failure and need for consecutive total hip arthroplasty (THA) [1]. Other studies demonstrated that typical fracture configurations of the elderly such as anterior column fractures, anterior wall fractures, anterior column posterior hemitransverse fractures, or both column fractures were associated with fair to poor clinical and radiological results in more than 30% of cases [5]. Especially superior-medial dome impaction of the acetabulum is predictive for fixation failure in elderly patients [4]. For open reduction and internal fixation of the medialized quadrilateral plate, the ilioinguinal approach has been established [6]. Due to the relevant morbidity of the ilioinguinal approach, the modified Stoppa approach was developed as a less invasive surgical access [7]. While the ilioinguinal approach exposes the pelvic brim under direct visualization except for the quadrilateral plate and the acetabular dome, which may result in a suboptimal reduction of impacted acetabular dome fractures or displaced quadrilateral plate, the modified Stoppa approach allows direct view under the pelvic brim including the quadrilateral plate [8]. Alternatively, with respect to potential neurological and vascular complications or to peritoneal breach, the pararectus approach has been developed as alternative access directly to the joint which combines the advantages of the second and third windows of the ilioinguinal approach with the medial view of the modified Stoppa approach to treat acetabular fractures with affection of the quadrilateral plate [9]. But only few studies on functional outcome exist in the literature, and cohort groups are comparatively small resulting in a strong need for further clinical data [10, 11].

This retrospective analysis of prospectively collected data presents functional mid-term results 2 years after open reduction and internal fixation of displaced acetabular fractures involving the anterior column using the pararectus approach.

## Materials and methods

**Fig. 1:**
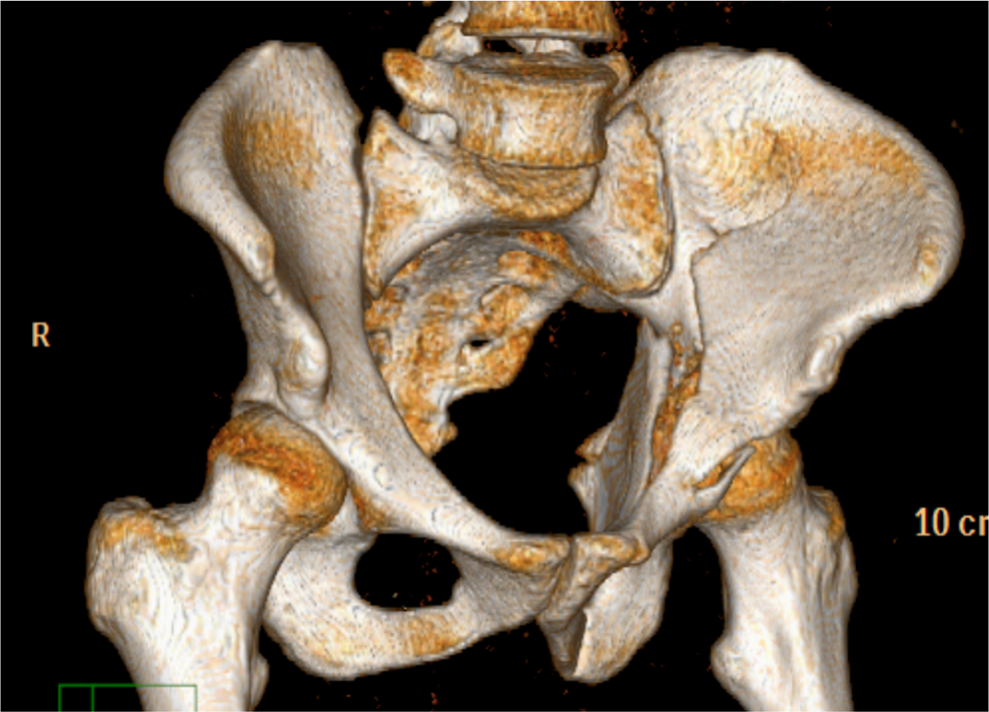
Both column acetabular fracture with dislocation of the quadrilateral plate and impaction of the acetabular dome

Between July 2012 and February 2016, 52 patients (12 female, 40 male) with a median age of 55 (range 18–90) years were treated in a single level I trauma centre suffering transverse, anterior column, anterior column posterior hemitransverse, both column and T-shaped acetabular fractures. All patients were treated surgically using the pararectus approach as a single surgical approach. Fractures were assessed preoperatively using CT scans (Fig. 1) and classified according to the Judet and Letournel classification as described previously [1, 12]. Inclusion criteria contained acute fractures less than 14 days after trauma, patients presenting with comminuted acetabular fractures involving the anterior column, and patients finally followed up 24 months after surgery. Exclusion criteria included patients younger than 18 years, patients suffering concomitant femoral head fractures, isolated posterior wall fractures, or bilateral acetabular fractures, as well as patients with fracture-related nerve damage, with pre-existing ipsilateral hip disease or with skeletal immaturity, and patients unable to give a written informed consent for the study.

### Surgical technique

**Fig. 2:**
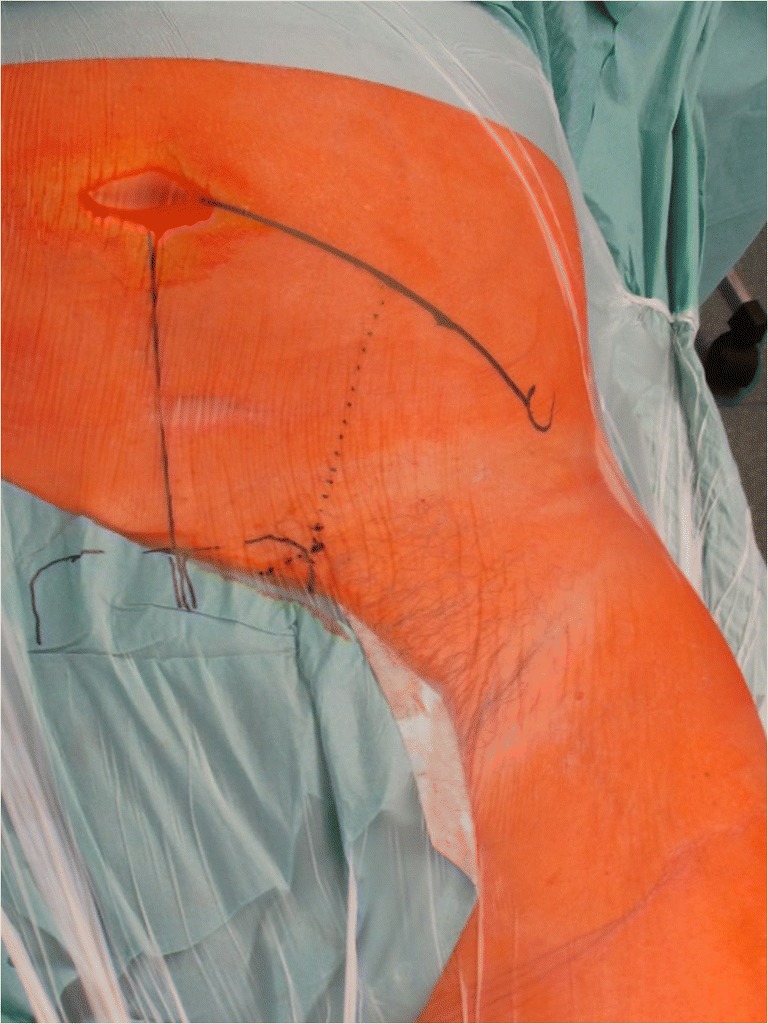
Surgical access using the pararectus approach: the incision (dotted line) starts cranially at the junction of the lateral and middle thirds of the line connecting the umbilicus with the anterior superior iliac spine. The incision ends at the border between the middle and medial thirds of the line connecting the anterior superior iliac spine with the symphysis. If necessary, an extension of the incision is possible (extended dotted line)

**Fig. 3:**
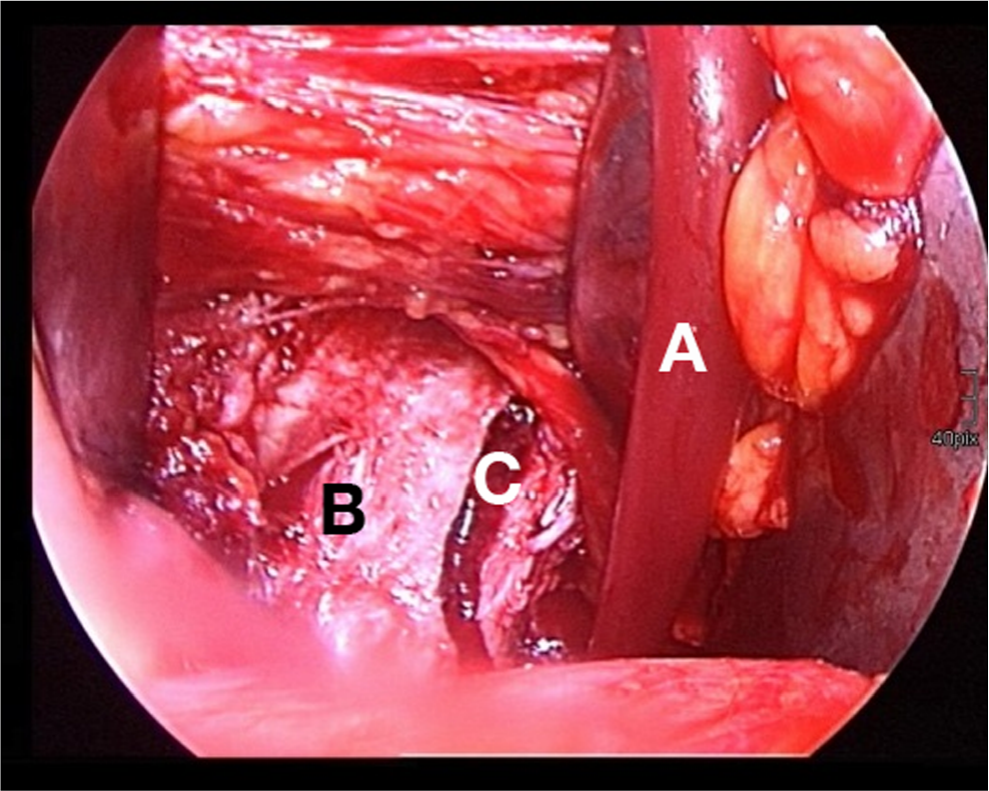
Retraction of mobilized external iliac vessels (A) provides optimal visualization of the pelvic brim and the quadrilateral plate (B). The direct intraoperative view into the fracture gap (C) facilitates anatomical fracture reduction

All surgical interventions and instrumentations were precisely performed by the same team of experienced senior surgeons in the same hospital according to the reports by Keel et al. [9, 13]: Skin incision started cranially at the junction of the lateral and middle thirds of the line connecting the umbilicus with the anterior superior iliac spine (Fig. 2). The incision ended at the border between the middle and medial thirds of the line connecting the anterior superior iliac spine with the symphysis. After dissection of the rectus sheath, the rectus abdominis muscle was mobilized and retracted medially. The extraperitoneal space was entered by incision of the transversalis fascia in a longitudinal direction. Laterally, the iliopsoas muscle appeared. Caudomedially, the neurovascular structures and the vas deferens respectively the round ligament were protected. The bladder and the obturator vessels were retracted medially. Retraction of mobilized external iliac vessels provided visualization of the quadrilateral plate. The direct intraoperative view into the fracture gap (Fig. 3) developed through the pararectus approach clearly facilitates anatomical fracture reduction and correct positioning of the small fragment plate.

The identical rehabilitation protocol was conducted with all patients. Physical therapy started immediately, and patients were allowed for toe-touch weight-bearing during the first two weeks post-operatively. Afterwards, partial weight-bearing with a maximum of 20 kg was allowed for another four  weeks. Antithrombotic prophylaxis using low molecular weight heparin was provided daily until full weight-bearing was achieved.

### Follow-up

**Fig. 4:**
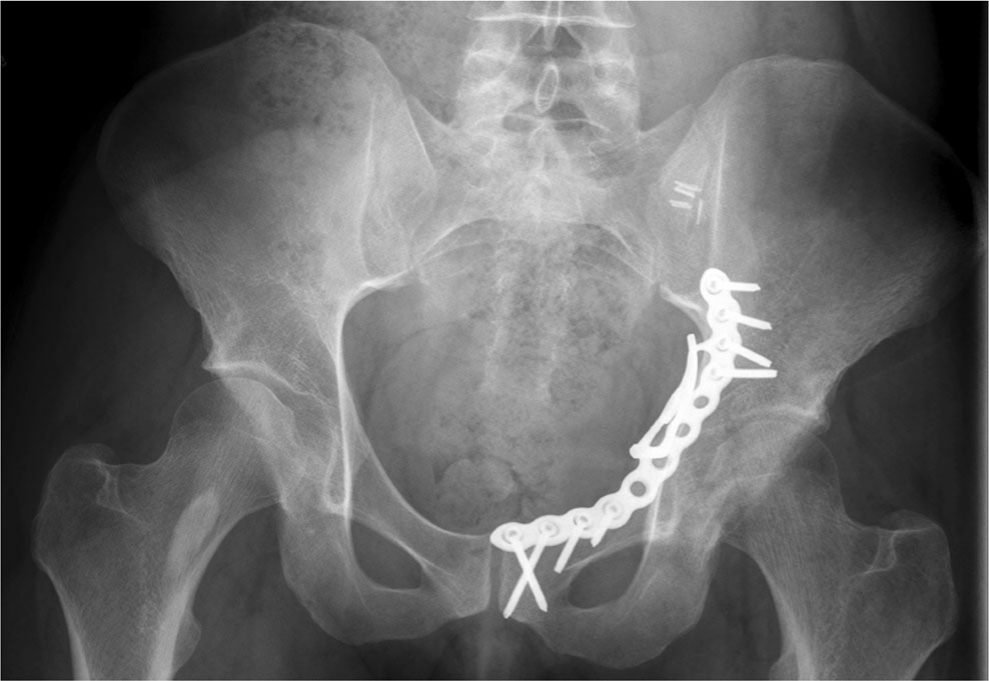
Post-operative X-ray demonstrates the fracture reduced anatomically using a small fragment plate (Stryker PRO system, Stryker Corp., Kalamazoo, MI, USA) on the pelvic brim and quadrilateral plate without any step or gap

Surgical data such as range of time from trauma to surgery, duration of surgical intervention and treatment in hospital, and intra-operative and post-operative complications were documented. Follow-up studies were performed at regular intervals including three, six, 12, and 24 months post-operatively. Final functional follow-up assessment was accompanied by diagnostic X-rays (Fig. 4) and CT scans. Fracture healing was defined using the following clinical and radiological outcome parameters: ability to perform weight bearing without pain, stability at fracture site, and the elimination of fracture lines at the level of the weight bearing dome [14]. Quality of fracture reduction was assessed according to Matta criteria [15]. The influence of treatment outcome on patients’ subjective and objective health status was assessed using the modified Merle d’Aubigné score, the Lower Extremity Functional Scale, the Western Ontario and McMaster Universities Osteoarthritis Index (WOMAC), and the 36-Item Short Form Survey (SF-36).

### Statistical analysis

Dependent to the amount of observations, results in this study are presented as mean and standard deviation (SD) or median values and quartiles. Data was managed using Microsoft Excel 2010 (Microsoft Corp., Redmond, WA, USA).

### Compliance with ethical standards

All procedures performed in this study were in accordance with the ethical standards of the institutional and the national review boards (Ethics Committee of the Bavarian State Chamber of Physicians, approval number: 16043) and with the 1964 Helsinki declaration and its later amendments.

The study was conducted according to ICMJE guidelines and has been retrospectively registered with the German Clinical Trials Register (approval number: DRKS00011308).

## Results

Fifty-two patients with comminuted acetabular fractures surgically treated using the pararectus approach were included. An overview on patients’ demographic and peri-operative data is presented in Table 1. Thirty patients suffered the acetabular fracture from polytrauma. Two severely injured patients died during clinical course due to multiple organ failure, but not related to the acetabular fracture, and were excluded. Median range of time between trauma and definite surgical treatment was three (range 0–19) days. Fractures were fixed using 3.5-mm small fragment reconstruction plates and cortical screws (Stryker Corp., Kalamazoo, MI, USA; DePuy Synthes Companies, Zuchwil, Switzerland). All surgical incisions healed by primary intention. Median operation time was 140 (range 60–240) minutes, and duration of hospital treatment was 19 (range 7–38) days. According to the modified Matta criteria, reduction was anatomical in 85% of cases in post-operative CT scans. None of the operations resulted in major intra-operative complications. Minor vascular lesions occurred in two patients and were managed intra-operatively without any consequences in the further clinical course. Post-operative complications are presented in Table 2. Six patients with a median age of 72 years developed post-traumatic osteoarthritis, resulting in THA within the observation period.

**Table 1:** Patients’ demographic and peri-operative data overview

Parameter	Value	Percent
Male	40	64.5
Female	12	35.5
Age* [years]	55 (18–90)	
Age > 60 years	23	44.2
Mechanism of injury		
Car accident	13	25
Motor bike accident	4	7.7
Bicycle accident	8	15.4
Fall > 3 m	5	9.6
Fall < 3 m	18	34.6
Base jump accident	1	1.9
Skiing accident	3	5.8
Monotrauma	22	42.3
Polytrauma	30	57.7
Judet and Letournel classification		
Both column	22	42.4
Anterior column	6	11.5
Transverse	2	3.8
Anterior column posterior hemitransverse	16	30.8
T-shaped	6	11.5
Delay to surgery* [days]	3 (0–19)	
Operation time* [minutes]	140 (60–240)	
Duration of hospital treatment* [days]	19 (7–38)	

*Results are presented as median

**Table 2:** Post-operative complications

Complication	Patients [*n*]
Subcutaneous hematoma	1
Superficial wound infection	1
Obturator nerve affection	1
Pelvic deep vein thrombosis	2
Implant breakage	1

Thirty-four patients were available for final follow-up 24 months after revision surgery, among them eight females and 26 male patients with a median age of 58 (range 20–85) years. Nineteen patients suffered from polytrauma (without traumatic brain injury), while the remaining 15 patients demonstrated an isolated acetabular fracture. Range of time between trauma and surgical treatment in these 34 patients was two (range 1–12) days. Median operation time was 140 (range 80–220) minutes, and median duration of hospital treatment was 21 (range 10–38) days. Thirty out of these 34 fractures (88%) healed within 12 months after surgery. Functional outcome was excellent in 22 patients (65%), good in eight patients (23.5%), and fair in four patients (11.5%) according to the modified Merle d’Aubigné score (excellent: 18 points, good: 15–17 points, fair: 14 or 13 points, poor: < 13 points; median: 16 points; range 13–18 points), 68 points (median; range 39–80) according to the Lower Extremity Functional Scale (maximum: 80 points), 6% according to the WOMAC (mean; SD ± 14.5%) (maximum: 96 points), and 69% (mean; SD ± 20.1%) according to the SF-36 (maximum: 100 points) (Table 3).

**Table 3:** Functional results according to Lower Extremity Functional Scale, modified Merle d’Aubigné score, WOMAC, and SF-36 24 months post-operatively in 34 patients

Score	Results
Lower Extremity Functional Scale*	68 points (range 39–80)
WOMAC**	6% (± 14.5)
SF-36**	69% (± 20.1)
Modified Merle d’Aubigné score*	16 points (range 13–18)
	Excellent (18 points): 22 patients (65%)
	Good (15–17 points): 8 patients (23.5%)
	Fair (14 or 13 points): 4 patients (11.5%)

*Median; **Mean +/SD

## Discussion

The ilioinguinal approach has been considered for a long time as the standard anterior approach for acetabular fractures involving the anterior column [1]. Recently, the modified Stoppa approach became more and more important due to its better visualization of the intrapelvic anatomy including the quadrilateral plate [6]. Furthermore, the pararectus approach has been introduced to treat fractures mainly involving the anterior column and the quadrilateral plate with good access to fracture site, adequate visualization of essential neurovascular structures, feasibility for anatomical restoration even in cases with impaction of the superomedial joint surface, and minimally invasive soft-tissue dissection [9].

In dislocated acetabular fractures, the articular surface should be reduced anatomically. Nevertheless, poor outcome has been reported in about 20% of all operated simple fractures and in nearly 30% of all operated comminuted fractures [5, 16]. In this regard, surgical outcome depends on fracture type, patients’ age, femoral head damage, delay to surgery, and quality of reduction [2, 17–19].

Dailey et al. mentioned that earlier surgical intervention improves the probability of achieving anatomical fracture reduction [17]. Median delay to surgery of three days in this study is similar to their results. The quality of fracture reduction depends on the adequate view of the fracture fragments. The results of the current study demonstrated similar reduction quality compared to earlier reports [11, 20]. In addition, Keel et al. reported an operation time of about 200 minutes using the pararectus approach [9, 13]. In the current study, operation time decreased to a median of 140 minutes which is equal to or even quicker than the reported operation time for latest described minimally invasive surgical techniques [21, 22]. Besides, Keel et al. demonstrated good or excellent clinical results after a follow-up period of at least two years post-operatively in 94% of patients [11]. For the use of the modified Stoppa approach, other studies reported good to excellent clinical outcomes in 69 to 89% for comminuted acetabular fractures involving the anterior column [6, 7, 23–25]. The current study demonstrated 88% good to excellent functional results in the examined patients for comparable fracture patterns using similar functional grading systems such as modified Merle d’Aubigné score which is the most widely accepted functional hip sore evaluating the clinical result of acetabular fracture treatment [11, 26, 27].

Surgical complications such as hernia, thrombosis, neurovascular injuries, and hematoma are seen at rates of approximately 10% for the ilioinguinal approach [28] and are comparable to rates of 11% for the pararectus approach in this study. Furthermore, 11.5% of patients with median age of 72 years obtained THA in terms of post-traumatic osteoarthritis. In addition, with 23 patients over 60 years, the results of the current study confirm Keel’s findings that the pararectus approach seems to be a feasible approach also for elderly patients with anterior or central destruction of the acetabulum [10, 11]. This is of particular importance since the incidence of acetabular fractures including involvement of the anterior column is constantly increasing [4] and has more than doubled from the early 1990s to 2010 [29]. In this regard, anterior column fractures and anterior column posterior hemitransverse fractures are the most common fracture patterns [30, 31]. In the German pelvis registry, an increase from 3 to 19% within 20 years could be observed [32]. In the current study, anterior column with or without posterior hemitransverse fractures accounted for approximately 42% of all fractures.

In general, innovations in surgery often are criticized in terms of missing transferability of commonly good follow-up results of the initial authors to a frequent number of users. For the use of the pararectus approach, this study confirms the good to excellent functional results of the describer. Therefore, regardless of patients’ age, the pararectus approach has become the standard approach in our institution for initial surgical treatment of acetabular fractures involving the anterior column or central joint fragments.

### Rationales for the use of the pararectus approach

The pararectus approach enables excellent exposition of the anterior wall, the anterior column, and the quadrilateral plate. It facilitates sufficient view and preparation of the dome fragment through the displaced quadrilateral plate or an additional little window by using an osteotomy at the innominate line. Additionally, additive screw placement through the medial plate is an option. Further dorsal preparation enables complete presentation of the anterior part of the sacroiliac joint which might be helpful in combined fractures including displacement of the sacroiliac joint [13]. Limitations are seen in high-riding fractures of the anterior column. In these fractures, the first window laterally to the iliopsoas muscle can be displayed through the pararectus approach or through a little additional incision at the iliac crest [33].

Although a potential disadvantage of the pararectus approach is seen in the risk for a peritoneal lesion while it is located between peritoneum and lateral abdominal muscles, it therefore provides a direct medial view to fracture fragments. In case of peritoneal lesions, the peritoneum can be easily sued and closed. Injury of intra-abdominal structures has not been reported in the literature and was not present in our study [34].

A main advantage of the surgical technique performed in this study is to reduce fracture fragments against the direction of the fracture displacement. In the standard anterior approaches mainly tension and compression without direct visualization are used to reduce fracture fragments. This facilitates anatomical reduction and leads to higher quality of fracture retention without fracture gap. Thereby, loose intra-articular fracture fragments can be removed, the cartilage can be evaluated, and impacted dome fragments can be reduced anatomically under direct view. Furthermore, the approach eases medial plate positioning and enables to counteract permanently against centrally directed fracture forces, which biomechanically improves the stability of the osteosynthesis. In addition, direct visualization of the fracture can be improved by flexion of the leg and deep anesthesia providing enough muscle relaxants.

### Study limitations

The limitations of this study include the retrospective evaluation of prospectively collected data, the missing control group, the limited number of patients available for final follow-up, and the consecutive lost to follow-up of 34% which is mainly based on the relatively high amount of polytraumatized patients treated in our level I trauma centre [35]. The variety of patients’ age as well as the sequential nature of the cohort group, the different range of time between trauma and definite surgical treatment, and the missing recordings of intra-operative blood loss are limiting factors. On the other hand, all patients were managed with a standard treatment protocol in the same hospital by the same team of experienced surgeons.

## Conclusions

Based on the results of this study, the pararectus approach can be considered as an equal if not superior alternative in surgical treatment of acetabular fractures involving the anterior column, independent of patients’ age. Intra-operatively, the pararectus approach enables good visualization of fracture fragments allowing secure anatomical reduction. Further prospective multi-centric studies are intended to confirm these considerations in larger cohorts.

## Abbreviations

WOMAC: Western Ontario & McMaster Universities Osteoarthritis Index
SF-36: 36-Item Short Form Survey
SD: standard deviation
THA: Total hip arthroplasty
